# Profiling of Cerebrospinal Fluid Lipids and Their Relationship with Plasma Lipids in Healthy Humans

**DOI:** 10.3390/metabo11050268

**Published:** 2021-04-24

**Authors:** Kosuke Saito, Kotaro Hattori, Shinsuke Hidese, Daimei Sasayama, Tomoko Miyakawa, Ryo Matsumura, Megumi Tatsumi, Yuuki Yokota, Miho Ota, Hiroaki Hori, Hiroshi Kunugi

**Affiliations:** 1 Division of Medical Safety Science, National Institute of Health Sciences, Kanagawa 210-9501, Japan; 2 Department of Mental Disorder Research, National Institute of Neuroscience, National Center of Neurology and Psychiatry, Tokyo 187-8502, Japan; hattori@ncnp.go.jp (K.H.); shidese@ncnp.go.jp (S.H.); sasayama@shinshu-u.ac.jp (D.S.); tmiyakawa@ncnp.go.jp (T.M.); megumi_tatsumi@ncnp.go.jp (M.T.); y_yokota@ncnp.go.jp (Y.Y.); ota@md.tsukuba.ac.jp (M.O.); hori@ncnp.go.jp (H.H.); 3 Medical Genome Center, National Center of Neurology and Psychiatry, Tokyo 187-8551, Japan; rmatsumura@ncnp.go.jp; 4 Department of Psychiatry, Teikyo University School of Medicine, Tokyo 173-8605, Japan

**Keywords:** cerebrospinal fluid, lipidomics, lipid profiling, mass spectrometry, plasma lipid

## Abstract

Lipidomics provides an overview of lipid profiles in biological systems. Although blood is commonly used for lipid profiling, cerebrospinal fluid (CSF) is more suitable for exploring lipid homeostasis in brain diseases. However, whether an individual’s background affects the CSF lipid profile remains unclear, and the association between CSF and plasma lipid profiles in heathy individuals has not yet been defined. Herein, lipidomics approaches were employed to analyze CSF and plasma samples obtained from 114 healthy Japanese subjects. Results showed that the global lipid profiles differed significantly between CSF and plasma, with only 13 of 114 lipids found to be significantly correlated between the two matrices. Additionally, the CSF total protein content was the primary factor associated with CSF lipids. In the CSF, the levels of major lipids, namely, phosphatidylcholines, sphingomyelins, and cholesterolesters, correlated with CSF total protein levels. These findings indicate that CSF lipidomics can be applied to explore changes in lipid homeostasis in patients with brain diseases.

## 1. Introduction

Lipidomics provide an overview of lipid profiles in biofluids and tissues [1,2,3]. The biological functions of lipids are diverse and are associated with multiple processes, including apoptosis, inflammation, proliferation, and differentiation [4,5,6]; thus, lipid profiling is a potential strategy to better understand disease pathophysiology and explore biomarkers. Indeed, blood lipid profiling is used to assess several diseases, including cancer, diabetes, and Alzheimer’s disease [7,8,9]. Although blood, in the form of plasma and/or serum, is commonly used for lipid profiling in humans, cerebrospinal fluid (CSF) is the biological fluid with the closest association with the brain, containing molecules of neural cell origin. Hence, the CSF reflects the overall status and activity of the brain and is, therefore, particularly attractive for those investigating biomarkers of brain diseases [10,11]. There are several CSF biomarkers for brain disorders, for example, tau, phosphorylated tau, and Aβ42 can be used for the diagnosis of Alzheimer’s disease with a high (~90%) sensitivity and accuracy [12]. Meanwhile, anti-N-methyl-d-aspartate (NMDA) receptor encephalitis, which often causes psychosis resembling schizophrenia, can be diagnosed by the presence of anti-NMDA receptor antibodies in the CSF [13]. Thus, CSF provides a more suitable matrix for exploring lipid homeostasis in patients with brain diseases.

CSF lipid profiling can provide insights into the pathophysiology of brain diseases, such as multiple sclerosis, Alzheimer’s disease, and amyotrophic lateral sclerosis, as well as to screen for biomarkers for such diseases. High levels of CSF ceramides are associated with multiple sclerosis [14], elevated diacylglycerols and phosphatidylethanolamines (PEs), as well as decreased plasmalogen-type phosphatidylethanolamines (PEps) were detected in Alzheimer’s patients [15], while high levels of ceramides and glucosylceramides (CerG1s) were found in patients with amyotrophic lateral sclerosis [16]. Therefore, fundamental information regarding CSF lipid profiles must be disseminated. Although plasma lipid levels are known to differ based on different factors, such as sex, age, body mass index (BMI), and diet, it remains unclear whether the overall individual background of each person affects the CSF lipid profile. For example, plasma sphingomyelins (SMs) are higher in females than in males, whereas plasma triacylglycerols (TGs) are higher in older females than in young females [17]. Another study, which demonstrated that plasma SM levels are higher in females, also showed that cholesterolesters (ChEs) and TGs are positively associated with BMI, whereas lysophospholipids are negatively associated [18]. Moreover, the plasma levels of several phosphatidylcholines (PCs) are associated with dietary carbohydrate-fat ratios [19]. In addition to each person’s individual background, specific molecular properties are critical for CSF–plasma correlations. For instance, the close correlation of substance P levels between CSF and plasma can enable the evaluation of headaches associated with varying plasma substance P levels [20]. In contrast, plasma and CSF tau levels are poorly correlated in Alzheimer’s disease [21]. Nevertheless, the association between CSF–plasma and lipid profiles in healthy individuals is yet to be evaluated.

The current study collected demographic information, as well as CSF and plasma samples, from 114 healthy subjects, and a lipidomics platform was used to analyze their lipid profiles. First, the CSF and plasma lipid profiles were compared and potential correlations were examined. Subsequently, the effects of CSF properties and background of the subjects on CSF lipids were evaluated. This study provides fundamental information regarding CSF lipid profiles, and on changes in CSF lipids due to the CSF condition and the individual background of each person. Cumulatively, these findings may represent a guideline for future CSF application for biomarker screening via lipidomics.

## 2. Results

### 2.1. CSF Profiles in Human Subjects

The present study enrolled 114 healthy subjects, from whom CSF and plasma lipid profiles were obtained (*n* = 114 and 99, respectively). The CSF properties assessed included: neutrophil, lymphocyte, and red blood cell counts, as well as protein, glucose, and chloride concentrations. The information regarding subjects was not limited to age and sex, but also included allergies, smoking and alcohol drinking habits, postprandial time, height, and BMI, although some of these data were missing for some of the participants. Demographic and lifestyle-related information of the participants is shown in Tables S1 and S2. Briefly, the age range was from 19 to 75 years (median 40 years) and a slight majority of the participants were male (54%). CSF protein concentration ranged between 20 to 72 mg/dL (median 36 mg/dL). A total of 19 of 58 participants reported an allergic experience, 11 of 83 were smokers, and 41 of 58 had the habit of drinking alcohol. The range of postprandial time (*n* = 93) was 0.5 to 18 h (median 2.33 h), the height range (*n* = 63) was from 146.6 to 180.1 cm (median 165 cm), and the BMI range (*n* = 63) was 17.72 to 32.46 (median 21.65).

**Figure 1:**
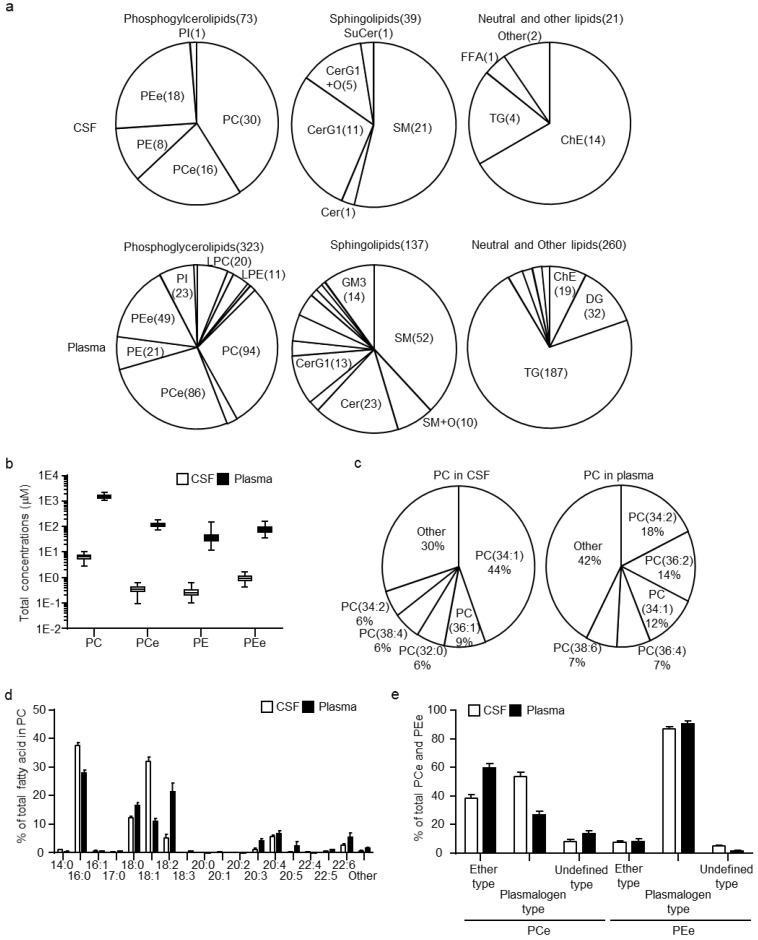
Cerebrospinal fluid (CSF) and plasma lipid profiles in healthy human subjects. (**a**) Diagram of the ratio of each lipid class calculated using the number of individual lipids within the class in phosphoglycerolipids, sphingolipids, and neutral lipids. (**b**) Estimated total concentration of phosphatidylcholine (PC), ether-type PCs (PCe), phosphatidylethanolamine (PE), and ether-type PEs (PEe) in CSF and plasma. (**c**) Diagram of the molar ratio of PCs calculated using the estimated concentrations of individual PCs within the total PC concentrations. (**d**) Fatty acid levels within PCs. Abundance of each fatty acid as percentage of all side chains, calculated as the ratio of the sum of concentrations containing the fatty acid to 2× the sum of peak heights of all PCs. (**e**) Compositions of ether-type and plasmalogen-type within PCes and PEes. The molar ratios of ether-type and plasmalogen-type were calculated using the estimated sum concentrations of ether-type and plasmalogen-type within the total PCe or PEe concentrations. Data are shown mean ± standard deviation.

Lipidomic analysis resulted in quantification of 133 and 720 lipids in CSF and plasma, respectively (Table S1). The estimated concentrations of PCs and ether-type PCs (PCes), which also included plasmalogen-type PCs (PCps), as well as of PEs and ether-type PEs (PEes), including plasmalogen-type PEs (PEps), in the CSF and plasma, were determined. In addition, other arbitrary lipid units normalized using PC internal standards were assessed. The composition of individual lipid numbers within classes of phosphoglycerolipids, sphingolipids, and neutral, as well as other lipids are illustrated in Figure 1a. In the case of phosphoglycerolipids, the lysophosphatidyl glycerolipids, such as lysophosphatidylcholines, lysophosphatidylethanolamines, and phosphatidylinositols, had a lower composition ratio in CSF than in plasma. For sphingolipids, the composition ratios of CerG1 and oxidized CerG1 (CerG1+O) were higher, whereas those of oxidized SMs (SM+O), ceramides, and ganglioside GM3s were lower in CSF than in plasma. For neutral and other lipids, the composition ratio of ChEs was higher, whereas those of diacylglycerols and TGs were lower in CSF than in plasma. In addition, the sum concentrations of PCs, PCes, PEs, and PEes were 234-, 332-, 147-, and 84-fold lower, respectively, in CSF than in plasma (Figure 1b). When the mol% ratios of PCs (the most abundant lipid class in the CSF) were compared, the estimated concentrations of the top five major PCs were clearly different between CSF and plasma (Figure 1c). The major PC in CSF was PC(34:1), which accounted for 44% of all PCs, followed by PC(36:1), PC(32:0), PC(38:4), and PC(34:2). In contrast, the major PC in the plasma was PC(34:2), which accounted for 18% of all PCs, followed by PC(36:2), PC(34:1), PC(36:4), and PC(38:6). Determination of each fatty acid side chain in PCs by fragment analysis showed that the fatty acids (FA)(16:0) and FA(18:1) in PCs were abundant in CSF, whereas the FA(18:2), FA(20:3), FA(20:5), and FA(22:6) were scarce (Figure 1d). Since plasmalogens, PCps and PEps, are lipids well-known to be associated with brain function [22], PCes and PEes were classified into actual ether-types (PCes and PEes) and plasmalogen-types (PCps and PEps). The mol% ratio of PCps was much higher in the CSF than in the plasma and accounted for more than 60% of the PCes (Figure 1e). In contrast, PEps were more predominant than PEes in both CSF and plasma, and did not significantly differ between the samples.

### 2.2. CSF–Plasma Correlations of Lipids

**Figure 2:**
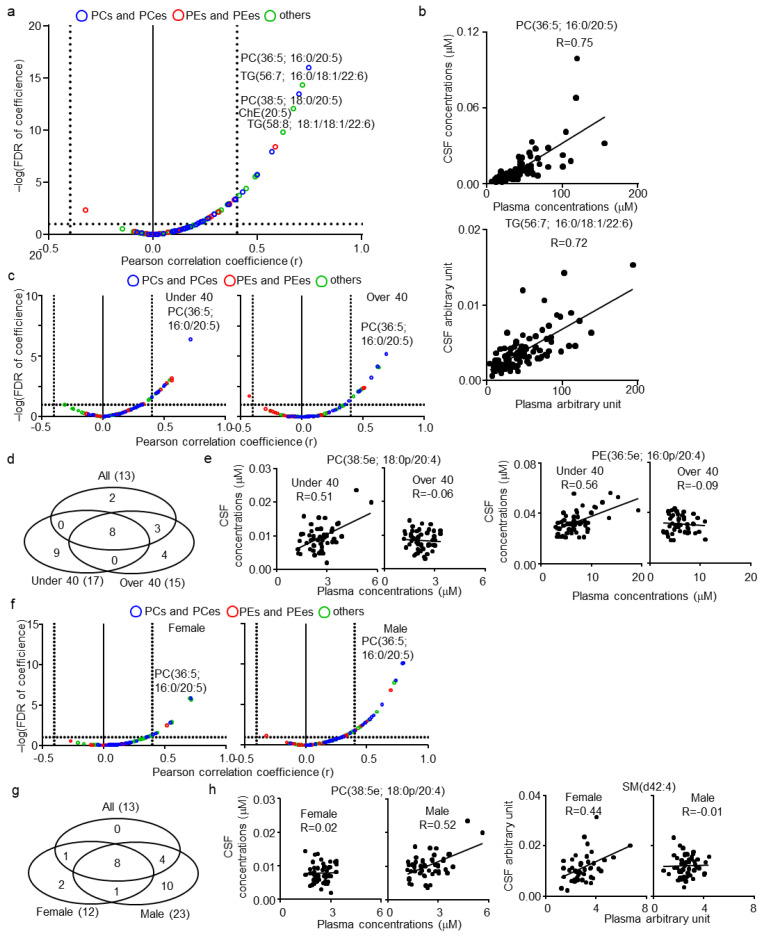
Cerebrospinal fluid (CSF)–plasma correlations of lipids in healthy human subjects. (**a**) Individual lipid plot of false discovery rate (FDR) and correlation coefficients (r) in all subjects. (**b**) Individual plot of representative lipids correlated among CSF and plasma. (**c**) Individual lipid plot of FDR and correlation coefficients in ≤40 (younger group) and >40 (older group) years-old subjects. (**d**) Venn diagram of correlated lipids in all, younger, and older subjects. (**e**) Individual plot of age-specific representative lipids correlated among CSF and plasma. (**f**) Individual lipid plot of FDR and r in female and male subjects. (**g**) Venn diagram of correlated lipids in all, female, and male subjects. (**h**) Individual plot of sex-specific representative lipids correlated among CSF and plasma.

Following the comparison of lipid profiles of CSF and plasma, the correlation between lipids of the CSF and plasma in healthy human subjects was evaluated next. Of the 133 CSF lipids, 114 were also quantified in plasma and subjected to analysis. The list of lipids and their correlation coefficient (r) and statistical values (p and false discovery rate (FDR)) are summarized in Table S3. As a result, 13 lipids were defined as CSF–plasma correlated lipids (|r| > 0.4; FDR < 0.1), of which 10 contained highly polyunsaturated FA; FA(20:5), FA(22:5), or FA(22:6) (Figure 2a). The correlations of representative lipids, PC(36:5; 16:0/20:5) and TG(56:7; 16:0/18:1/22:6) between CSF and plasma are shown in Figure 2b. Along with the correlation in the total subjects, the effect of age and gender on the correlation between CSF and plasma lipids were also assessed. To compare the age-associated correlation between CSF and plasma lipids, the subjects were divided into two groups: ≤40 years (younger group) and >40 years (older groups) (median split). The subjects in the two groups presented similar trends in the correlation between CSF and plasma lipids (Figure 2c). Clustering of lipids defined as CSF–plasma correlated lipids of all, younger and older subjects demonstrated that nine and five were specific to the younger and older group, respectively (Figure 2d). Of the CSF–plasma correlated lipids specific to the younger group, six demonstrated delta r values over 0.4 (Table S3). The delta r was calculated by subtracting the r in the younger group from the r in the older group. In addition, all six lipids were PCps and PEps, of which five contained FA(20:4). The correlation of representative lipids, PC(38:5e; 18:0p/20:4) and PE(36:5; 16:0p/20:4), is shown in Figure 2e. Female and male subjects also presented a similar trend in the correlation between CSF and plasma lipids, but males showed a higher coefficient (Figure 2f). Clustering of lipids defined as CSF–plasma correlated lipids in all, female and male subjects demonstrated that two and 10 were specific for females and males, respectively (Figure 2g). Of the CSF–plasma correlated lipids specific for males and females, only one (SM(d42:4)) in females and four (PC(36:3; 18:1/18:2), PC(38:3; 18:0/20:3), PC(38:5e; 18:0p/20:4), and PE(38:5e; 16:0p/22:4) in males demonstrated delta r values over 0.4 (Table S3). The correlation of representative lipids, PC(38:5e; 18:0p/20:4) and SM(d42:4), is shown in Figure 2h.

### 2.3. CSF–Plasma Correlations of Lipids

**Figure 3:**
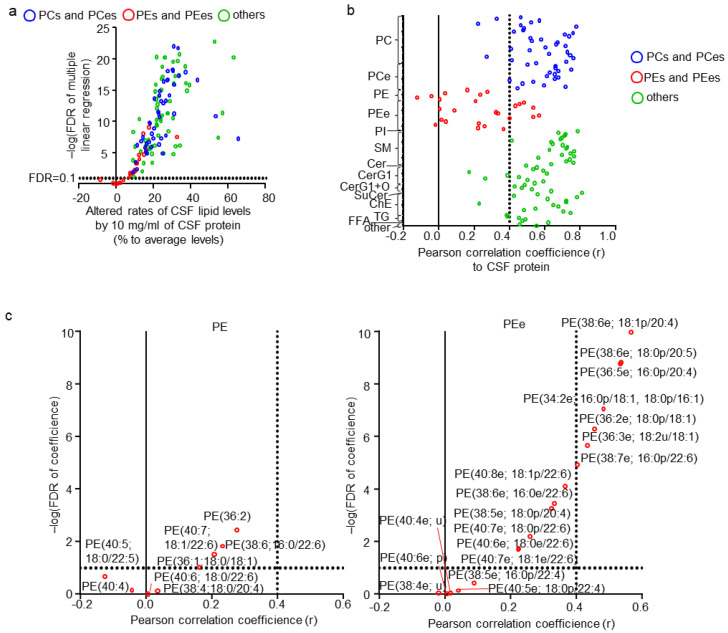
Effects of cerebrospinal fluid (CSF) properties on CSF lipids. (**a**) Individual lipid plot of false discovery rate (FDR) and estimated beta corresponding to 10 mg/mL of CSF total protein levels. (**b**) Individual lipid plot of correlation coefficients to CSF total protein levels. (**c**) Extracted individual lipid plot of FDR and correlation coefficients of phosphatidylethanolamine (PEs) and ether-type PEs (PEes).

Next, the effect of CSF properties (such as CSF neutrophil, lymphocyte, red blood cell count, total protein, glucose, and chloride levels) on CSF lipid levels were examined. None of the combinations of these CSF properties were correlated (|r| < 0.4) (Table S4). Therefore, each property was evaluated as an individual parameter. To evaluate the complex effects of multiple factors, the multiple regression model was used, and beta, p, and FDR were estimated (Table S5). Of the total quantitated CSF lipids, 124 (93.2%) were linearly associated with CSF total protein levels (Figure 3a; FDR < 0.1), whereas no lipid exhibited an FDR < 0.1 for all other CSF properties. Interestingly, all lipids that were not associated with CSF total protein levels were PEs and PEes. The plot of the correlation coefficients for CSF protein and lipid levels clearly demonstrated such a trend (Figure 3b). Although 28 lipids, including three PCs, eight PEs, 11 PEes, three CerG1s, one ChE, one ChE(C28), and one TG, were not defined as being correlated with CSF total protein levels (|r| < 0.4), only four PEs and five PEes exhibited an FDR > 0.1 (Table S6). Individual plots of lipids in PEs and PEes also showed trends of contained FA side chains with the correlation of CSF protein and lipid levels (Figure 3c). PEs containing FA(18:0), such as PE(38:4; 18:0/20:4) and PE(40:6; 18:0/22:6), showed lower correlation coefficients and a higher FDR than others, such as PE(40:7; 18:2/22:6) and PE(38:6; 16:0/22:6). In contrast, PEes containing FA(22:4), such as PE(40:5e; 18:0p/22:4) and PE(38:5e; 16:0p/22:4), followed by FA(22:6), showed lower correlation coefficients and a higher FDR than others, such as PE(38:6e; 18:1p/20:4) and PE(38:6e; 18:0p/20:5).

### 2.4. Effects of Subject Background on CSF Lipids

**Figure 4:**
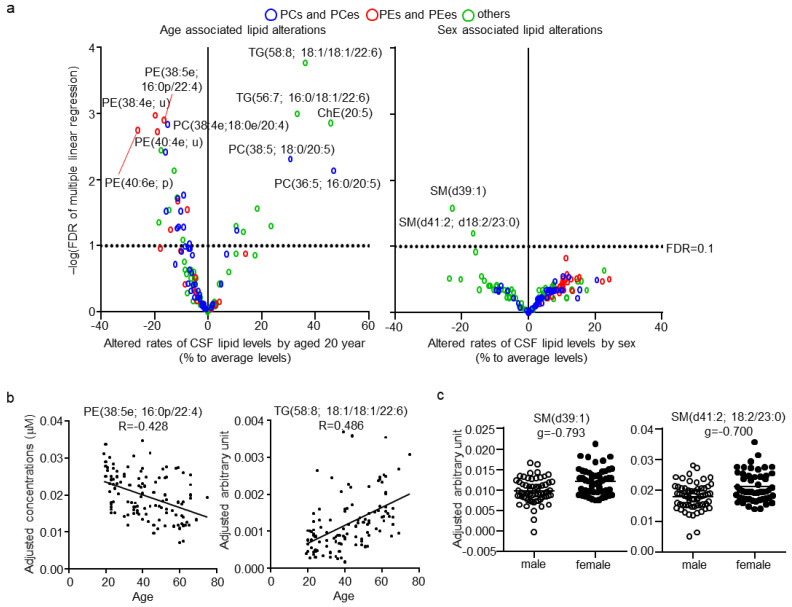
Effects of age and sex on cerebrospinal fluid (CSF) lipids. (**a**) Individual lipid plot of false discovery rate (FDR) and estimated beta corresponding to 20 years of age and sex. (**b**) Individual plot of representative lipids correlated with age. (**c**) Individual plot of representative lipids differing between males and females.

Next, the effects of age and sex, which are major factors affecting lipid levels in plasma, on CSF lipids were evaluated. No correlation was found (|r| < 0.4) between CSF total protein levels, age, and sex (Table S7). CSF total protein levels, age, and sex were evaluated independently, using a multiple regression model and estimated beta, p, and FDR for these three factors (Table S8). Of the total quantitated CSF lipids, 33 and two (24.8% and 1.5%, respectively) were associated with age and sex, respectively (Figure 4a; FDR < 0.1). Ten lipids were positively associated with age, eight of which contained the highly unsaturated FA; FA(20:5), FA(22:5), or FA(22:6). In contrast, 23 lipids were negatively associated with age, with 15 of them being PCes and PEes, and of those nine were PCps and PEps. The representative correlation plot of lipids TG(58:6; 16:0/18:1/22:6) and PE(38:5e; 16:0p/22:4), adjusted for the other two factors, are shown in Figure 4b. The lipids associated with sex were both SMs with odd numbered carbon chains, namely SM(d39:1) and SM(d41:2; d18:2/23:0). The individual plot of these lipids, adjusted for the other two factors, are shown in Figure 4c.

**Figure 5:**
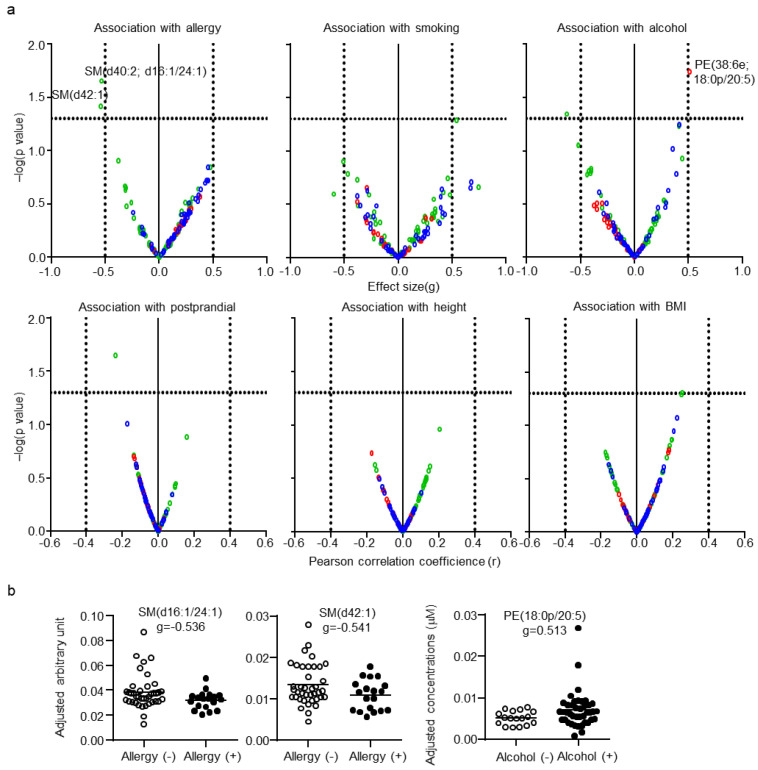
Effects of the background of the subjects on cerebrospinal fluid (CSF) lipids. (**a**) Individual lipid plot of false discovery rate (FDR) and effect size or correlation coefficients. (**b**) Individual plot of representative lipids altered by allergy and alcohol habits.

After adjustment for CSF total protein levels, age, and sex, the effects of subject background on CSF lipid levels were determined. Since the information regarding the other subject backgrounds was random in some subjects, these factors were examined individually. Each categorical parameter was evaluated using effect size, whereas each numerical parameter was evaluated using correlation coefficients. Only three lipids, SM(d40:2; d18:1/24:1), SM(d42:1) for allergy, and PE(38:6e; 18:0p/20:5) for alcohol habit, were defined as being associated with the other subject backgrounds (Figure 5a). The individual plot of these lipids with adjustment are shown in Figure 5b. No significant association of any CSF lipid with smoking, postprandial effect, height, or BMI was found.

## 3. Discussion

The current study comprehensively demonstrated the profiles of CSF lipids and their association with plasma lipids in healthy subjects. A key finding of the present study was revealed in the comparison of CSF and plasma lipid profiles of the same subjects. The levels of CSF lipids were found to be far less than those of plasma lipids, and the composition of CSF lipids was clearly different from that of plasma lipids. For example, the FA side chains of PCs differed between CSF and plasma, wherein PCps were found in much higher abundance in the CSF than in plasma. In addition, only a limited number of lipids of the CSF and plasma were correlated; some of these were specific for age and sex. FA(22:6), corresponding to docosahexaenoic acid (DHA), and FA(20:5), corresponding to eicosapentaenoic acid (EPA), containing lipids were generally correlated, whereas correlations of several plasmalogens were specific for younger group subjects and males. Furthermore, the factors associated with CSF lipid levels were characterized. CSF total protein level was found to be the dominant factor regulating CSF lipid levels, except for several PEs and PEes, with over 90% of CSF lipid levels being associated with CSF total protein levels. Age was also a major factor regulating CSF lipid levels. DHA and EPA containing lipids were positively associated with age, whereas ether lipids, including plasmalogens, were negatively associated with age. Other factors, such as sex and allergy, were not associated with most CSF lipids, although several SMs were enriched in female and allergy-negative subjects.

DHA is highly enriched in the brain and is essential for brain development and function [23,24]. EPA, a polyunsaturated fatty acid present at lower levels in the brain, has similar influence. Evidently, DHA and EPA deficiencies are associated with the emergence of neurological diseases [25]; therefore, DHA and EPA play critical roles in brain function. To date, several mechanisms have been proposed as responsible for transporting DHA from the plasma to the brain, of which one major mechanism is the Mfsd2a-dependent transport using lysophosphatidylcholine incorporated DHA [26,27]. In the present study, DHA and EPA containing lipids, such as PC(36:5; 16:0/20:5) and TG(56:7; 16:0/18:1/22:6), of the CSF and plasma were associated, and such an association was not affected by age or sex. Since CSF is in close contact with the brain and contains molecules of neural cell origin, these results suggest that the underlying mechanism transporting DHA and EPA containing lipids from the plasma to the brain is active in healthy individuals and maintains brain function regardless of age and sex. In addition to CSF–plasma correlation, a positive correlation of DHA and EPA containing lipids in plasma with age was reported [17], which was confirmed in the present study (Figure S1). Thus, age-associated differences in levels of DHA and EPA containing lipids in CSF could be attributed to the plasma-CSF transport mechanism.

Plasmalogens represent another class of important lipids required for brain function [22,28]. Decreased plasma plasmalogen levels were reported in brain diseases such as Alzheimer’s disease, depression, and bipolar disease [15,29,30]. The current study demonstrated that, unlike DHA and EPA containing lipids, several correlated plasmalogens in the CSF and plasma, such as PC(38:5e; 18:0p/20:4) and PE(36:5e; 16:0p/20:4), were specific to younger subjects and, partly, to males. This finding suggests that the mechanism underlying the transport of plasmalogens from the plasma to the brain is more active in young individuals and in some males. This may be relevant to the fact that elderly individuals, and particularly females, have a higher risk for Alzheimer’s disease [31]. Thus, plasmalogen transport from the plasma to the brain may be one of the factors regulating the development of plasmalogen-dependent brain diseases. The age-dependent lower levels of CSF plasmalogens, such as PE(38:5e; 16:0p/22:4) and PE(40:6e; p), are associated with a decrease in the supply of plasmalogens to the brain in the elderly. Currently, the underlying mechanism remains to be fully characterized, although the low-density lipoprotein receptor-mediated transcytosis pathway was suggested to be involved [32]. Therefore, characterization of the main mechanism underlying the correlation between several plasmalogens of the CSF and plasma may provide a good clinical target for the treatment of plasmalogen-dependent brain diseases.

The characterization of CSF total protein levels as the dominant factor of CSF lipid levels is another feature of the present study. Levels of global classes of lipids, except for several PEs and PEes, are positively associated with CSF protein levels. The major CSF protein reported in healthy subjects was serum albumin, which accounts for over 50% of all proteins [10,33]. Reportedly, serum albumin forms complexes with PCs and lysophosphatidylcholines [34,35]. Thus, CSF total protein levels associated with lipids, at least in part, form complexes with albumin and are, therefore, associated with albumin levels in healthy subjects. The present study also demonstrated that CSF protein levels in healthy subjects varied 3.6-fold (20–76 mg/dL) and were not correlated with age and sex. These findings also suggest that CSF total protein levels should be taken into consideration when screening for CSF biomarkers using lipidomics, although brain diseases were shown to modulate the composition of CSF total protein levels [36,37], thereby implying that the association trend among protein and lipid levels may not be consistent among healthy subjects and patients with brain diseases. In addition, brain diseases, such as multiple sclerosis and glioma, were found to increase CSF total protein levels [38,39], which were believed to be associated with greater permeability of the blood-brain-barrier by its disruption. In fact, it is widely accepted as the gold standard that blood-brain-barrier permeability is evaluated by calculation of the CSF-serum albumin quotient [40,41]. Therefore, it is also possible that the levels of global classes of CSF lipids are strongly affected by blood-brain-barrier permeability.

The present study has several limitations. First, although self-reported healthy subjects who had not taken any medication prior to at least one week before study start were recruited, asymptomatic disease was inevitable. In addition, the diet of the participant was not controlled, which may have affected the plasma and CSF lipid levels, although the limited postprandial effect on CSF lipids was in agreement with a previous study [42]. Furthermore, certain aspects of information related to the background of some subjects, such as allergies and smoking habits were lacking, making such data unsuitable for multiple regression analysis with CSF protein levels, age, and sex. Therefore, the effects of CSF total protein levels, age, and sex were adjusted to examine the effect of such data on CSF lipid levels. This approach may have resulted in false positives or negatives, although the subjects included in each comparison numbered over 50 in total. Lastly, we applied 40 years of age as the median split to examine age-associated differences, despite the age of onset of amyloid-beta accumulation in Alzheimer’s disease and that of degenerative brain diseases occurring after 40 years [43,44]. In the literature, the menopausal effect of plasma lipid profiles has been previously demonstrated [45,46]. However, although the menopausal effect on the CSF lipid profile is also an interesting point, we could not conduct the analysis because of a dearth of menopausal information from the participants. In addition, a larger scale study is warranted to verify the findings of the present study.

## 4. Materials and Methods

### 4.1. Subjects and Sample Collection

Subjects were recruited at the National Center of Neurology and Psychiatry, Tokyo, Japan, through a local free magazine or our website announcement. All participants were biologically-unrelated Japanese individuals and underwent a structured interview using the Mini-International Neuropsychiatric Interview, Japanese version [47,48], administered by trained psychologists or psychiatrists. Individuals with current or a history of major psychiatric illness, history of central nervous system disease, severe head injury, substance abuse, with severe medical illness, or expectant women were excluded from the study.

CSF samples were obtained by lumbar puncture as described previously [48]. Briefly, following neurologic examinations, each participant received local skin anesthesia followed by a lumbar puncture at L3–4 or L4–5 using an atraumatic pencil point needle (Unilever 22G, 75 mm, Unisis Corp., Tokyo, Japan). The initial 2 mL of CSF obtained was used for laboratory tests, including cell counts, and total protein and glucose concentrations. The next 8–10 mL of CSF was collected into a low-protein adsorption tube (PROTEOSAVE SS 15 mL conical tube, Sumitomo Bakelite Co., Tokyo, Japan) and immediately chilled on ice. The CSF was centrifuged (4000× *g*, 10 min, 4 °C) and the supernatant was dispensed into 0.5 mL aliquots in low-protein adsorption tubes (PROTEOSAVE SS 1.5 mL Slimtube, Sumitomo Bakelite Co.) and stored in a deep freezer (−80 °C). For lipidomics, the samples were thawed once and re-dispensed into 120 μL aliquots in smaller low-protein adsorption tubes (PROTEOSAVE SS 0.5 mL Slimtube, Sumitomo Bakelite Co.) and stored in a deep freezer (−80 °C) before use.

Simultaneously, blood samples were collected via venipuncture into 7 mL EDTA-2Na-containing vacuum blood collection tubes (VENOJECT II, Terumo, Tokyo, Japan). The samples were immediately centrifuged (2500 × *g*, 10 min, 4 °C), dispensed in screw-capped polypropylene tubes (96 Jacket Tubes 1.3 mL internal type, FCR&Bio Co., Ltd., Japan), and stored in a deep freezer (−80 °C). For lipidomics, the samples were thawed once and re-dispensed into 120 μL aliquots in polypropylene tubes (96 Jacket Tubes 1.3 mL internal type, FCR&Bio Co.) and stored at −80 °C before use.

This study was conducted in accordance with the Declaration of Helsinki and approved by the Ethics Committee of the National Center of Neurology and Psychiatry (no. A2019-092) and the National Institute of Health Sciences (no. 278). Written informed consent was obtained from all participants.

### 4.2. Lipidomics

Lipid extraction was performed using a Microlab NIMBUS workstation (Hamilton, Binaduz, Switzerland). CSF samples were mixed with 1 volume of methanol containing 2 μM of an internal standard (PC[1 2:0/12:0] and PE[12:0/12:0]), followed by 3 volumes of 1% formic acid in water. The mixed samples were then subjected to solid phase extraction with FastRemover C18 (GL Science, Tokyo, Japan) and MPE2 unit (Hamilton). The preparations were washed with water and 50% methanol, and then subjected to elution with methanol and isopropanol. The resulting lipid-containing solution was evaporated and reconstituted with 30 μL of methanol for lipidomics. Plasma samples were mixed with 9 volumes of methanol:isopropanol (1:1) containing 2 μM of internal standard (PC[12:0/12:0]). Mixed samples were filtered with FastRemover Protein (GL Science) using an MPE2 unit. The resulting lipid-containing solution was directly subjected to lipidomics.

To obtain lipidomic data, we performed reversed-phase liquid chromatography (Ultimate 3000, Thermo Fisher Scientific, Waltham, MA, USA) and MS (Orbitrap Fusion, Thermo Fisher Scientific), following a recently established lipidomics platform [49]. An InertSustainSwift C18 column (3 μm, 2.1 × 250 mm [P]; GL Science) was used and the temperatures of the column oven and sample tray were set to 55 and 4.5 °C, respectively. The mobile phase was pumped at a flow rate of 250 μL/min. Mobile phase A was composed of water:methanol:acetonitrile (21:20:60 [*v*:*v*:*v*]) with 0.1% formic acid and 10 mM ammonium formate, and the mobile phase B was composed of water:acetonitrile:isopropanol (1:10:90 [*v*:*v*:*v*]) with 0.1% formic acid and 10 mM ammonium formate. A multistep gradient was used as follows: the gradient was initiated at 10% solvent B and subsequently increased to 40% solvent B over 5 min. The mobile phase was further increased to 50% solvent B between 5 and 10 min, and finally changed to 100% between 10 and 18 min, prior to maintaining it at 100% mobile phase B for further 4 min. The column was equilibrated with 10% mobile phase B for 5 min before the next sample was injected.

Compound Discoverer 2.0 (CD 2.0; Thermo Fisher Scientific) was used with the raw data for peak extraction, annotation, identification, and lipid quantification, as described previously [50]. Full MS data were used for quantification, and MS2 and MS3 data were used for qualification of lipids. Lipid annotation by CD 2.0 was determined using a mass list of monoisotopic ion mass calculated with a formula. Identification of annotated lipids was conducted using our internal library that was developed by comparison with standards or specific MS2/MS3 fragments, as per our previous reports [49]. If isomers were identified (same annotation of class, carbon length, and number of double bonds), a distinct letter was placed after the class of each lipid molecule to distinguish the isomers. The quantified raw data were normalized to internal standards. Since the reverse phased lipidomics platform demonstrated similar ionization efficacy among lipid classes regardless of fatty acid side chains, the concentrations of different PCs classes, including PC, PCe, and lysophosphatidylcholines, were determined using PC(12:0/12:0) as internal standard, while concentrations of PEs classes, including PE, PEe, and lysophosphatidylethanolamines, were determined using PE(12:0/12:0) as internal standard. The processed data for the lipid levels are presented in Table S1.

### 4.3. Statistical Analysis

Significant differences in individual lipid levels between groups were assessed using FDR, except for the effect of allergy, alcohol consumption, smoking, postprandial effect, height, or BMI, which were assessed by *p*-values of unpaired Student’s *t*-test. FDR was calculated using *p*-values of Student’s *t*-tests for comparisons between groups and *p*-values of multiple regression model. The multiple regression analysis was conducted using R 3.6.0 software (R Foundation for Statistical Computing, Vienna, Austria). In this study, a lipid level was considered significantly different if its FDR was <0.1.

## 5. Conclusions

The current study revealed and characterized detailed fundamental lipid profile of CSF in humans. As CSF is the biofluid with the closest association with the brain, this information is useful for exploring changes in lipid homeostasis in patients with neuropsychiatric disorders.

## Data Availability

All datasets are provided as Supplementary Tables. The whole raw data will be published in April 2025.

## Supplementary Materials

The following are available online at https://www.mdpi.com/article/10.3390/metabo11050268/s1, Figure S1: Individual plot of representative plasma lipids correlated with age, Table S1: Demographic and lipidomics data set obtained in the present study, Table S2: Summarized demographic information of subjects recruited in the present study, Table S3: CSF–plasma correlation of lipids in healthy human subjects, Table S4: Correlations of CSF properties used in the present study, Table S5: Calculated beta and *p*-values of CSF properties in multiple linear regression model, Table S6: Correlation of CSF lipids and CSF protein levels in healthy human subjects, Table S7: Correlations of CSF protein, sex, and age, Table S8: Calculated beta and *p*-values of CSF protein, sex, and age in multiple linear regression model, Table S9: Statistical values of the effects of subjects’ background on CSF lipid levels.

## References

[1] Houjou T., Yamatani K., Imagawa M., Shimizu T.R., Taguchi R. (2005). A shotgun tandem mass spectrometric analysis of phospholipids with normal-phase and/or reverse-phase liquid chromatography/electrospray ionization mass spectrometry. Rapid Commun. Mass Spectrom..

[2] Han X., Gross R.W. (2005). Shotgun lipidomics: Electrospray ionization mass spectrometric analysis and quantitation of cellular lipidomes directly from crude extracts of biological samples. Mass Spectrom. Rev..

[3] Meikle P.J., Christopher M.J. (2011). Lipidomics is providing new insight into the metabolic syndrome and its sequelae. Curr. Opin. Lipidol..

[4] Mené P., Simonson M.S., Dunn M.J. (1989). Phospholipids in signal transduction of mesangial cells. Am. J. Physiol..

[5] Hannun Y.A., Linardic C.M. (1993). Sphingolipid breakdown products: Anti-proliferative and tumor-suppressor lipids. Biochem. Biophys. Acta.

[6] Fadok V.A., Bratton D.L., Rose D.M., Pearson A., Ezekewitz R.A., Henson P.M. (2000). A receptor for phosphatidylserine-specific clearance of apoptotic cells. Nature.

[7] Chen S., Yin P., Zhao X., Xing W., Hu C., Zhou L., Xu G. (2013). Serum lipid profiling of patients with chronic hepatitis B, cirrhosis, and hepatocellular carcinoma by ultrafast LC/IT-TOF MS. Electrophoresis.

[8] Zhao C., Mao J., Ai J., Shenwu M., Shi T., Zhang D., Wang X., Wang Y., Deng Y. (2013). Integrated lipidomics and transcriptomic analysis of peripheral blood reveals significantly enriched pathways in type 2 diabetes mellitus. BMC Med. Genom..

[9] Han X., Rozen S., Boyle S.H., Hellegers C., Cheng H., Burke J.R., Welsh-Bohmer K.A., Murali Doraiswamy P., Kaddurah-Daouk R. (2011). Metabolomics in early Alzheimer’s disease: Identification of altered plasma sphingolipidome using shotgun lipidomics. PLoS ONE.

[10] Hühmer A.F., Biringer R.G., Amato H., Fonteh A.N., Harrington M.G. (2006). Protein analysis in human cerebrospinal fluid: Physiological aspects, current progress and future challenges. Dis. Markers.

[11] Mouton-Barbosa E., Roux-Dalvai F., Bouyssié D., Berger F., Schmidt E., Righetti P.G., Guerrier L., Boschetti E., Burlet-Schiltz O., Monsarrat B. (2010). In-depth exploration of cerebrospinal fluid by combining peptide ligand library treatment and label-free protein quantification. Mol. Cell Proteom..

[12] Blennow K., Hampel H., Weiner M., Zetterberg H. (2010). Cerebrospinal fluid and plasma biomarkers in Alzheimer disease. Nat. Rev. Neurol..

[13] Dalmau J. (2016). NMDA receptor encephalitis and other antibody-mediated disorders of the synapse: The 2016 Cotzias Lecture. Neurology.

[14] Pieragostino D., Cicalini I., Lanuti P., Ercolino E., Di Ioia M., Zucchelli M., Zappacosta R., Miscia S., Marchisio M., Saccetta P. (2018). Enhanced release of acid sphingomyelinase-enriched exosomes generates a lipidomics signature in CSF of Multiple Sclerosis patients. Sci. Rep..

[15] Wood P.L., Barnette B.L., Kaye J.A., Quinn J.F., Woltjer R.L. (2015). Non-targeted lipidomics of CSF and frontal cortex grey and white matter in control, mild cognitive impairment, and Alzheimer’s disease subjects. Acta Neuropsychiatr..

[16] Blasco H., Veyrat-Durebex C., Bocca C., Patin F., Vourc’h P., Kouassi Nzoughet J., Lenaers G., Andres C.R., Simard G., Corcia P. (2017). Lipidomics reveals cerebrospinal-fluid signatures of ALS. Sci. Rep..

[17] Ishikawa M., Maekawa K., Saito K., Senoo Y., Urata M., Murayama M., Tajima Y., Kumagai Y., Saito Y. (2014). Plasma and serum lipidomics of healthy white adults shows characteristic profiles by subjects’ gender and age. PLoS ONE.

[18] Weir J.M., Wong G., Barlow C.K., Greeve M.A., Kowalczyk A., Almasy L., Comuzzie A.G., Mahaney M.C., Jowett J.B.M., Shaw J. (2013). Plasma lipid profiling in a large population-based cohort. J. Lipid Res..

[19] Inoue M., Senoo N., Sato T., Nishimura Y., Nakagawa T., Miyoshi N., Goda T., Morita A., Miura S. (2017). Effects of the dietary carbohydrate-fat ratio on plasma phosphatidylcholine profiles in human and mouse. J. Nutr. Biochem..

[20] Clark J.W., Senanayake P.D., Solomon G.D., Gallagher C. (1994). Substance P: Correlation of CSF and plasma levels. Headache.

[21] Fossati S., Ramos Cejudo J., Debure L., Pirraglia E., Sone J.Y., Li Y., Chen J., Butler T., Zetterberg H., Blennow K. (2019). Plasma tau complements CSF tau and P-tau in the diagnosis of Alzheimer’s disease. Alzheimers Dement..

[22] Braverman N.E., Moser A.B. (2012). Functions of plasmalogen lipids in health and disease. Biochim. Biophys. Acta.

[23] Lauritzen L., Brambilla P., Mazzocchi A., Harsløf L.B., Ciappolino V., Agostoni C. (2016). DHA Effects in brain development and function. Nutrients.

[24] Bos D.J., van Montfort S.J., Oranje B., Durston S., Smeets P.A. (2016). Effects of omega-3 polyunsaturated fatty acids on human brain morphology and function: What is the evidence?. Eur. Neuropsychopharmacol..

[25] Avallone R., Vitale G., Bertolotti M. (2019). Omega-3 fatty acids and neurodegenerative diseases: New evidence in clinical trials. Int. J. Mol. Sci..

[26] Lo Van A., Sakayori N., Hachem M., Belkouch M., Picq M., Lagarde M., Osumi N., Bernoud-Hubac N. (2016). Mechanisms of DHA transport to the brain and potential therapy to neurodegenerative diseases. Biochimie.

[27] Ben-Zvi A., Lacoste B., Kur E., Andreone B.J., Mayshar Y., Yan H., Gu C. (2014). Mfsd2a is critical for the formation and function of the blood-brain barrier. Nature.

[28] Su X.Q., Wang J., Sinclair A.J. (2019). Plasmalogens and Alzheimer’s disease: A review. Lipids Health Dis..

[29] Fujino T., Yamada T., Asada T., Tsuboi Y., Wakana C., Mawatari S., Kono S. (2017). Efficacy and blood plasmalogen changes by oral administration of plasmalogen in patients with mild Alzheimer’s disease and mild cognitive impairment: A multicenter, randomized, double-blind, placebo-controlled trial. EBioMedicine.

[30] Ogawa S., Hattori K., Ota M., Hidese S., Miyakawa T., Matsumura R., Yokota Y., Ishida I., Matsuo J., Yoshida S. (2020). Altered ethanolamine plasmalogen and phosphatidylethanolamine levels in blood plasma of patients with bipolar disorder. Psychiatry Clin. Neurosci..

[31] Ferretti M.T., Martinkova J., Biskup E., Benke T., Gialdini G., Nedelska Z., Rauen K., Mantua V., Religa D., Hort J. (2020). Sex and gender differences in Alzheimer’s disease: Current challenges and implications for clinical practice. Eur. J. Neurol..

[32] Candela P., Gosselet F., Miller F., Buee-Scherrer V., Torpier G., Cecchelli R., Fenart L. (2008). Physiological pathway for low-density lipoproteins across the blood-brain barrier: Transcytosis through brain capillary endothelial cells in vitro. Endothelium.

[33] Maccarrone G., Birg I., Malisch E., Rosenhagen M.C., Ditzen C., Chakel J.A., Mandel F., Reimann A., Doertbudak C.-C., Haegler K. (2004). In-depth analysis of the human CSF proteome using protein prefractionation. Clin. Proteom..

[34] Jonas A. (1976). Interaction of phosphatidylcholine with bovine serum albumin. Specificity and properties of the complexes. Biochim. Biophys. Acta..

[35] Kang J.X., Leaf A. (1996). Protective effects of free polyunsaturated fatty acids on arrhythmias induced by lysophosphatidylcholine or palmitoylcarnitine in neonatal rat cardiac myocytes. Eur. J. Pharmacol..

[36] Whitin J.C., Jang T., Merchant M., Yu T.T.-S., Lau K., Recht B., Cohen H.J., Recht L. (2012). Alterations in cerebrospinal fluid proteins in a presymptomatic primary glioma model. PLoS ONE.

[37] Link H. (1987). Contribution of CSF studies to diagnosis of multiple sclerosis. Ital. J. Neurol. Sci..

[38] Rohlff C. (2001). Proteomics in neuropsychiatric disorders. Int. J. Neurophyschopharmacol..

[39] Davidsson P., Sjögren M. (2005). The use of proteomics in biomarker discovery in neurodegenerative diseases. Dis. Markers..

[40] Andersson M., Alvarez-Cermeño J., Bernardi G., Cogato I., Fredman P., Frederiksen J., Frederiksen S., Gallo P., Grimaldi L.M., Grønning M. (1994). Cerebrospinal fluid in the diagnosis of multiple sclerosis: A consensus report. J. Neurol. Neurosurg. Psychiatry..

[41] Reiber H., Peter J.B. (2001). Cerebrospinal fluid analysis: Disease-related data patterns and evaluation programs. J. Neurol. Sci..

[42] Saito K., Hattori K., Andou T., Satomi Y., Gotou M., Kobayashi H., Hidese S., Kunugi H. (2020). Characterization of postprandial effects on CSF Metabolomics: A pilot study with parallel comparison to plasma. Metabolites.

[43] Bateman R.J., Xiong C., Benzinger T.L., Fagan A.M., Goate A., Fox N.C., Marcus D.S., Cairns N.J., Xie X., Blazey T.M. (2012). Clinical and biomarker changes in dominantly inherited Alzheimer’s disease. N. Engl. J. Med..

[44] Hou Y., Dan X., Babbar M., Wei Y., Hasselbalch S.G., Croteau D.L., Bohr V.A. (2019). Ageing as a risk factor for neurodegenerative disease. Nat. Rev. Neurol..

[45] Nogueira I.A.L., da Cruz É.J.S.N., Fontenele A.M.M., Figueiredo Neto J.A. (2018). Alterations in postmenopausal plasmatic lipidome. PLoS ONE.

[46] Beyene H.B., Olshansky G., Smith A.A.T., Giles C., Huynh K., Cinel M., Mellett N.A., Cadby G., Hung J., Hui J. (2020). High-coverage plasma lipidomics reveals novel sex-specific lipidomic fingerprints of age and BMI: Evidence from two large population cohort studies. PLoS Biol..

[47] Sheehan D.V., Lecrubier Y., Sheehan K.H., Amorim P., Janavs J., Weiller E., Hergueta T., Baker R., Dunbar G.C. (1998). The Mini-International Neuropsychiatric Interview (M.I.N.I.): The development and validation of a structured diagnostic psychiatric interview for DSM-IV and ICD-10. J. Clin. Psychiatry.

[48] Otsubo T., Tanaka K., Koda R., Shinoda J., Sano N., Tanaka S., Aoyama H., Mimura M., Kamijima K. (2005). Reliability and validity of Japanese version of the Mini-International Neuropsychiatric Interview. Pyschiatry Clin. Neurosci..

[49] Saito K., Ohno Y., Saito Y. (2017). Enrichment of resolving power improves ion-peak quantification on a lipidomics platform. J. Chromatogr. B Anal. Technol. Biomed. Life Sci..

[50] Saito K., Ikeda M., Kojima Y., Hosoi H., Saito Y., Kondo S. (2018). Lipid profiling of pre-treatment plasma reveals biomarker candidates associated with response rates and hand-foot skin reactions in sorafenib-treated patients. Cancer Chemother. Pharmacol..

