# Supplementary material for: Profiling of Cerebrospinal Fluid Lipids and Their Relationship with Plasma Lipids in Healthy Humans
Source: Metabolites. 2021 Apr 24;11(5):268. doi: 10.3390/metabo11050268 (PMC8146161; doi:10.3390/metabo11050268)
Supplement: Supplementary file 1 [file metabolites-11-00268-s001.zip › 210407 SupFigures CSF cohort.pptx]

## Slide 1
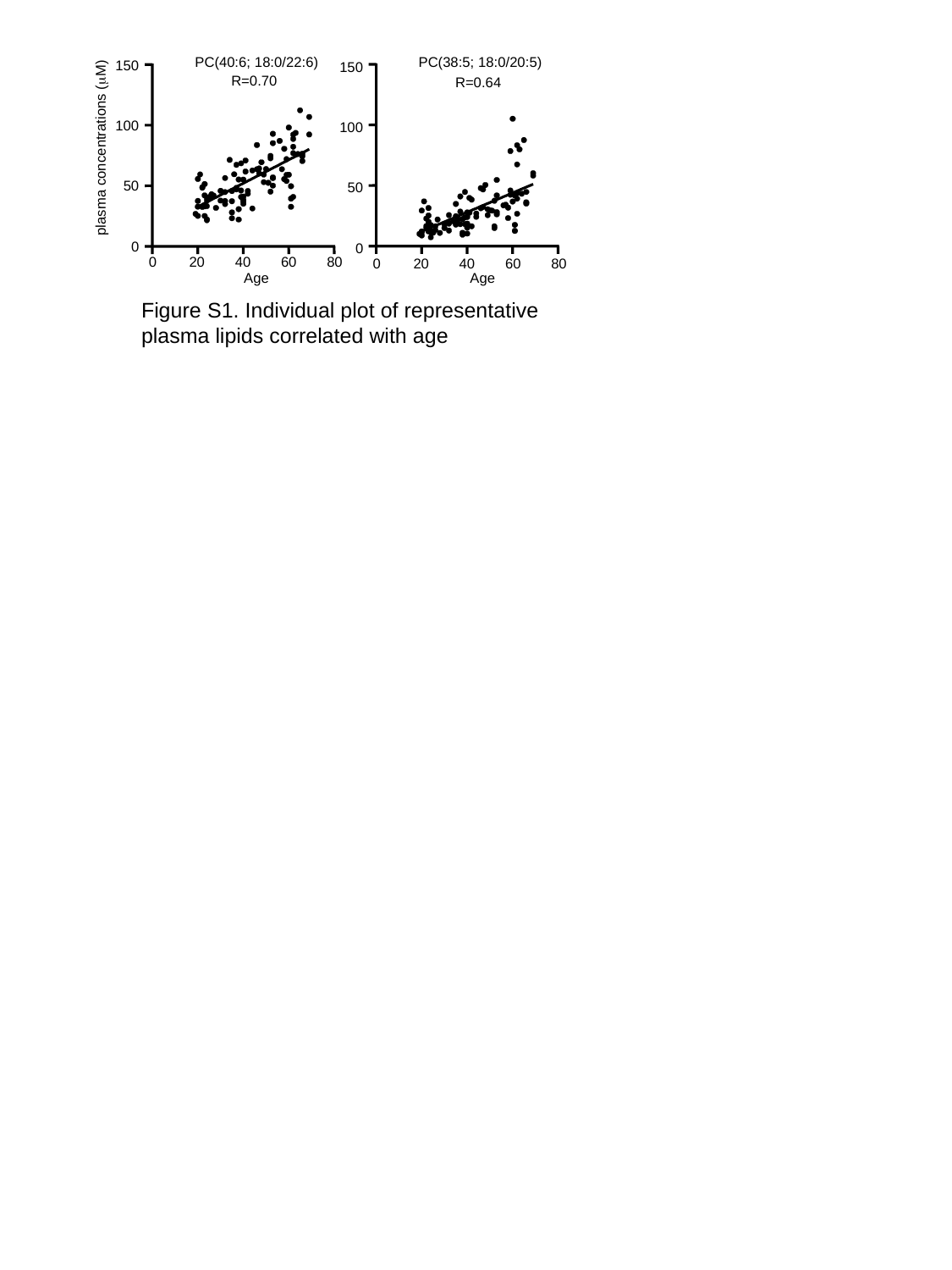

PC(40:6; 18:0/22:6)
PC(38:5; 18:0/20:5)
150
150
R=0.70
R=0.64
100
100
plasma concentrations (μM)
50
50
0
0
0
20
40
60
80
0
20
40
60
80
Age
Age
Figure S1. Individual plot of representative plasma lipids correlated with age
